# Patient and Family Determinants of Caregiver Burden in Major Depressive Disorder: A Multicenter Observational Study

**DOI:** 10.1192/j.eurpsy.2026.10615

**Published:** 2026-07-31

**Authors:** S. Cipolla, B. Della Rocca, A. Boiano, P. Catapano, M. Di Vincenzo, M. V. Lapadula, M. Gravagnone, C. Toni, M. Luciano, A. Fiorillo

**Affiliations:** Department of Psychiatry, University of Campania “Luigi Vanvitelli”, Naples, Italy

## Abstract

**Introduction:**

Approximately 60% of people living with mental health problems reside with their families. With the progressive shift to community-based psychiatric care, the closure of asylums, and the establishment of territorial services, families have become increasingly central in the management of severe mental disorders. While family involvement in major depressive disorder (MDD) is essential for patient recovery, the caregiving role itself critically affects the caregivers’ quality of life (QoL) and levels of stress. Family members of patients with schizophrenia or bipolar disorder often exhibit emotional overinvolvement, report high levels of subjective and objective burden, restrictions in social life, increased risk of depression/anxiety, financial difficulties, and reduced QoL. Furthermore, caregivers frequently experience powerlessness, hopelessness, and a sense of inability to change the situation.

**Objectives:**

The present study aims to identify both patient- and family-related factors influencing subjective and objective burden among relatives of patients with MDD. Moreover, we aim at identifying predictors for caregivers’ coping strategies and QoL.

**Methods:**

41 family units were recruited across 23 Italian centers. Each unit consisted of one patient with MDD and one or more relatives. Participants completed a set of questionnaires assessing socio-demographic and clinical characteristics, psychopathological conditions, and caregivers’ burden, coping strategies, and QoL. Regression analyses were performed by selecting independent variables significantly correlated with each dependent variable, adjusting for fixed factors.

**Results:**

Regression analyses highlighted several significant predictors of caregiver burden. In particular, objective family burden was associated with caregiver age (β = 0.17, p < 0.001), longer daily cohabitation with the patient (β = 0.17, p < 0.001), lower positive attitudes (β =−0.26, p < 0.001), criticism (β = 0.16, p < 0.01), and higher tolerance and expectation (β = 0.13, p < 0.01); patient-related predictors included the patient’s own objective burden (β = 0.22, p<0.001). Furthermore, caregiver’s gender (β = 0.17, p < 0.001), age (β = 0.19, p < 0.001), overinvolvement (β = 0.16, p < 0.001), criticism (β = 0.18, p<0.001) and lower positive attitudes (β = −0.163, p<0.001) was associated with increased caregiver subjective burden as well as patient-related variables such as subjective burden (β = 0.23, p < 0.001), criticism (β = 0.1, p < 0.05), sexual abuse (β = 0.1, p < 0.05) and CGI (β = 0.1, p < 0.05).

**Conclusions:**

The identification of specific determinants of higher family burden should serve as a warning for clinicians and highlight the importance of setting clear targets for family-oriented interventions. Psychoeducational strategies, in particular, may represent effective tools to support both families and patients, ultimately improving overall clinical and functional outcomes.

**Disclosure of Interest:**

None Declared

